# Supplementary material for: Clinical diversity and treatment results in Tegumentary Leishmaniasis: A European clinical report in 459 patients
Source: PLoS Negl Trop Dis. 2021 Oct 13;15(10):e0009863. doi: 10.1371/journal.pntd.0009863 (PMC8544871; doi:10.1371/journal.pntd.0009863)
Supplement: S1 Table — (A). Number of patients attended at each centre of the LeishMan consortium. (B). Number of cases according each country of LeishMan consortium. Abbreviations: UK, United Kingdom. (C). Suspected countries of acquisition in Old World. (D). Suspected countries of acquisition in New World. (E). Demographic and clinical characteristics of total cohort. Note. Results are expressed as number (%), unless otherwise stated. There are some missing data for each variable (<10%) explaining incomplete count proportion for categorical variables. Abbreviations: DissCL, disseminated cutaneous leishmaniasis; IQR, interquartile range; MCL, muco-cutaneous leishmaniasis, PKDL, post Kala-azar dermal leishmaniasis; VL, visceral leishmaniasis. (F). Subgenus, complex species, species identified in 198 cutaneous leishmaniasis infections. Note. *Viannia subgenus not further identified to species level. (DOC) [file pntd.0009863.s001.doc]

**S1 Table**

**Supplementary table A. Number of patients attended at each centre of the LeishMan consortium**

| **Centres** | **Cases, n (%)** |
| --- | --- |
| AMC Amsterdam | 38 (8) |
| BHH Birmingham | 1 (0·5) |
| CHU Rennes | 31 (7) |
| CNR Montpellier | 1 (0·5) |
| HTD London | 84 (18) |
| ITM Antwerpen | 12 (2) |
| ITMIH Berlin | 60 (13) |
| Necker-Pasteur Paris | 185 (40) |
| Public Health Agency of Sweden | 7 (1) |
| STPHI Basel | 45 (10) |
| **Total** | **464** |

**Supplementary table B. Number of cases according each country of LeishMan consortium**

| **Countries** | **Cases, n (%)** |
| --- | --- |
| France | 217 (47) |
| U.K | 85 (18) |
| Germany | 60 (13) |
| Switzerland | 45 (10) |
| Netherlands | 38 (8) |
| Belgium | 12 (2·5) |
| Sweden | 7 (1·5) |
| **Total** | **464** |

Abbreviations: UK, United Kingdom

**Supplementary table C. Suspected countries of acquisition in Old World**

| **Old World** | **Number of cases** |
| --- | --- |
| Spain | 48 |
| Syria | 36 |
| Morocco | 34 |
| Tunisia | 32 |
| Algeria | 22 |
| Senegal | 18 |
| Israel | 12 |
| France | 9 |
| Italy | 9 |
| Iran | 6 |
| Mauritania | 6 |
| Pakistan | 6 |
| Greece | 5 |
| Egypt | 4 |
| Sudan | 4 |
| Turkey | 4 |
| Mali | 3 |
| Afghanistan | 2 |
| Cyprus | 2 |
| Malta | 2 |
| Saudi Arabia | 2 |
| Eritrea | 1 |
| Ethiopia | 1 |
| Iraq | 1 |
| Libya | 1 |
| Namibia | 1 |
| Oman | 1 |
| Tanzania | 1 |
| Turkmenistan | 1 |
| United Arab Emirates | 1 |
| Unknown | 4 |
| **Total** | **279** |

**Supplementary table D. Suspected countries of acquisition in New World**

| **New World** | **Number of cases** |
| --- | --- |
| French Guiana | 41 |
| Peru | 37 |
| Costa Rica | 28 |
| Bolivia | 21 |
| Brazil | 15 |
| Belize | 9 |
| Ecuador | 7 |
| Mexico | 6 |
| Colombia | 3 |
| Suriname | 3 |
| Nicaragua | 2 |
| Panama | 2 |
| Argentina | 1 |
| French Caribbean | 1 |
| Guatemala | 1 |
| Guyana | 1 |
| Honduras | 1 |
| Unknown | 6 |
| **Total** | **185** |

**Supplementary table E. Demographic and clinical characteristics of total cohort**

| **Cutaneous leishmaniasis** | | | **464 episodes** |
| --- | --- | --- | --- |
| **Age, median [IQR]** | | | 30 [19-52] |
| **Male** | |  | 290 (62.5) |
| **Immunocompromised** | | | 21 (5) |
| **Previous history of leishmaniasis** | | | 34 (8) |
| **Categories of traveller** | | |  |
|  | Tourist | | 205 (44) |
|  | Visiting Friends and Relatives | | 129 (28) |
|  | Migrant / Soldier | | 39 (8) / 24 (5) |
|  | Others / Unknown | | 23 (5) / 15 (3) |
| **Type of cutaneous leishmaniasis** | | |  |
|  | Localized cutaneous | | 440 (95) |
|  | Muco-cutaneous | | 10 (2) |
|  | Mucosal | | 9 (2) |
|  | Rare forms (PKDL, DissCL, MCL + VL) | | 5 (1) |
| **Continent(s) of acquisition** | | |  |
|  | Old World / New World | | 279 (60) / 185 (40) |
| **Delay from first symptoms to the first consultation (months), median [IQR]** | | | 3 [2-6] |
| **Number of lesions, median [IQR]** | | | 2 [1-3] |
| **Type of lesions** | | |  |
|  | Ulcer (wet crust) | | 267 (58) |
|  | Papulo-nodular | | 79 (17) |
|  | Dry crust | | 70 (15) |
|  | Squamous plaque | | 28 (6) |
|  | Other | | 7 (2) |
|  | Scar | | 4 (1) |
| **Lesion localization** | | |  |
|  | Head / Neck and scalp | | 135 (30) / 10 (2) |
|  | Upper limb / Lower limb | | 142 (31) / 98 (21) |
|  | Trunk | | 19 (4) |
|  | Hands / Feet | | 36 (8) / 17 (4) |
| **Diameter of largest lesion (millimeter), median [IQR]** | | | 15 [10-30] |
| **Nodular lymphangitis** | | | 72/453 (16) |
| **Bacterial superinfection** | | | 19/238 (8) |

Note. Results are expressed as number (%), unless otherwise stated. There are some missing data for each variable (<10%) explaining incomplete count proportion for categorical variables.

Abbreviations : DissCL, disseminated cutaneous leishmaniasis; IQR, interquartile range; MCL, muco-cutaneous leishmaniasis, PKDL, post Kala-azar dermal leishmaniasis; VL, visceral leishmaniasis.

**Supplementary table F. Subgenus, complex species, species identified in 198 cutaneous leishmaniasis infections**

| **Identification** | **n** |
| --- | --- |
| *L. major* complex | 52 |
| *L. donovani* complex | 36 |
| *L. braziliensis* complex | 34 |
| *L. tropica* complex | 26 |
| Viannia subgenus* | 25 |
| *L. guyanensis* complex | 18 |
| *L. mexicana* complex | 4 |
| *L. lainsoni* | 1 |
| *L. martiniquensis* | 1 |
| *L. naiffi* | 1 |
| **Total** | **198** |
|  |  |

Note. *Viannia subgenus not further identified to species level.
