# Supplementary figures and images for: Clinical diversity and treatment results in Tegumentary Leishmaniasis: A European clinical report in 459 patients
Source: PLoS Negl Trop Dis. 2021 Oct 13;15(10):e0009863. doi: 10.1371/journal.pntd.0009863 (PMC8544871; doi:10.1371/journal.pntd.0009863)

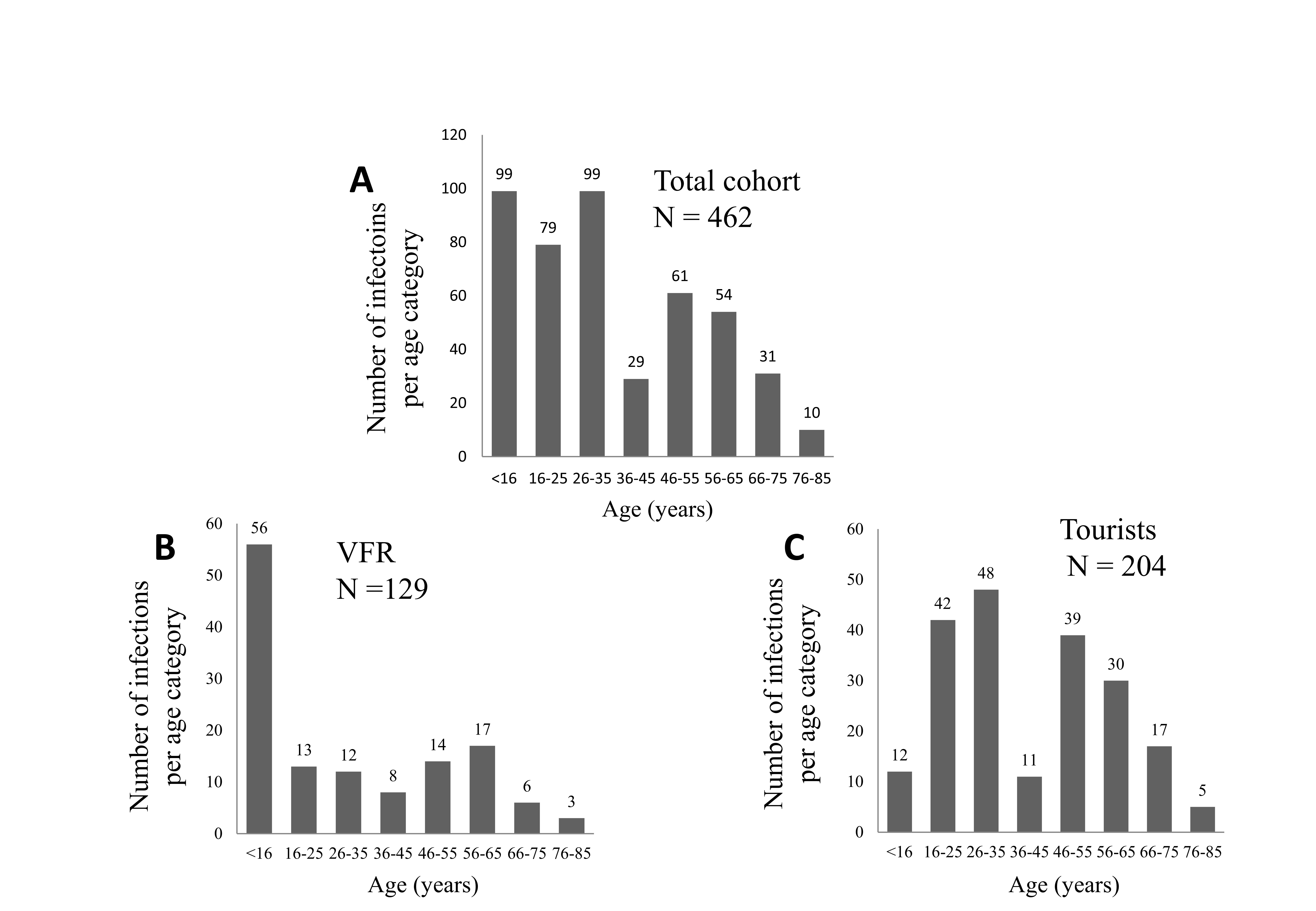

Supplement: S1 Fig — Panel A: age of distribution in total cohort; Panel B: age distribution in travellers visiting friends and relatives; Panel C: age distribution in tourists. Abbreviations: VFR visiting friends and relatives. (TIF) [file pntd.0009863.s002.tif]
