# Supplementary material for: Clinical diversity and treatment results in Tegumentary Leishmaniasis: A European clinical report in 459 patients
Source: PLoS Negl Trop Dis. 2021 Oct 13;15(10):e0009863. doi: 10.1371/journal.pntd.0009863 (PMC8544871; doi:10.1371/journal.pntd.0009863)
Supplement: Attachment — Submitted filename: 2020-05-21-response-to-reviewers.docx [file pntd.0009863.s005.docx]

Dear Editors

We are grateful for the accurate, specific and constructive comments provided by reviewers which markedly help improve the completeness, clarity and overall quality of the manuscript. You will find below our point-by-point answers to all questions and comments.

With respectful regards,

Romain GUERY, Diana LOCKWOOD, Johannes BLUM, Pierre BUFFET and the authors

**Response to Reviewer’s questions**

1. Methods 1

**Reviewer #1 and #2: No questions**

**Reviewer #3 “**It is a convenience cohort study, with 459 patients included, however the number of patients approached is not described in the methodology, only in the supplement file. Please make this information in the text.”

**Reviewer #4 “**2. A description of the loss of data for at least the therapeutic approach and parasite identification in the flow chart (figure 1), i.e., how many patients were analyzed in accordance of parasite species? and how many patients were included for the therapeutic approach comparison?”

Response: *We have amended Figure 1 according to the comments from Reviewer #3 and Reviewer #4 and included number of patients in whom species identification and therapeutic management analysis were performed (Please see below). To help readers navigation across the manuscript, we have added a reference to Tables & Figs corresponding to each analysis in Figure 1 (Changes are in blue).*

2. Methods 2 (Reviewer 4)

**The inclusion of the details of the multivariate approach for exploring the possible ML determinants in the method section, e.g. the criteria for inclusion of the variables in the final model (it is unusual just include de variables with p<0.05 significance level in the univariate analysis without other considerations related to the biological plausibility), the exploration of confusion and potential interactions between variables and the final evaluation of the model fitness.**

**Response :** *Despite the large size of the cohort the multivariate approach lacks indeed power. Conventionally, a logistic regression model should include at least 10 events per independent variable. We report 20 episodes of mucosal involvement, meaning that we should include only 2 variables in the model. We attempted to include more variables (5) with either statistical significance at the univariate step (age and immunocompromised status) or biological plausibility (gender, continent of acquisition). Our main objective was to confirm the lack of relationship between the continent of acquisition (i.e., Old versus New world) and risk of mucosal involvement, an important new descriptive observation of our report. However, we agree with the Reviewer that a multivariate analysis based on an even larger cohort would be more robust and thus may become the backbone of a future report. We have therefore removed the multivariate analysis for the manuscript. Nevertheless, and very importantly, we believe that, as it stands, the description of patients with mucosal involvement is already a solid source of carefully collected information that will help clinicians better grasp the risks and benefit of different management modalities of cutaneous leishmaniasis. Changes appear as follows:*

Analysis in the subgroup of patients with an identified *Leishmania* species found a prevalence of mucosal involvement of 22%, 5.8%, 0% and 0% for *L. (infantum)/donovani* Complex, *L. braziliensis/L. guyanensis* Complex*, L. tropica* Complex and *L. major* respectively **(Figure 3, Table 2)**. ~~Variables significantly associated with mucosal involvement at presentation on univariate analysis, namely, age > 50 years, sex, area (New World or Old World), immunosuppression~~ **~~(Table 3)~~** ~~were included in a multivariable conditional logistic regression model. According to this analysis, immunosuppression (OR 10.8 IC 95% [3.4–34]), age > 50 years (OR 4.8 IC 95% [1.7–14]), and male gender (OR 3.6 IC 95% [1.05–12.6]) were independently associated with mucosal involvement.~~

3. Methods 3 (Reviewer 2)

**Reviewer #1 : no questions**

**Reviewer #2 : 111: "...a previous history of leishmaniasis was reported by 8% of patients in the cohort". These were patients of CL with a previous episode of CL or ML patients with a previous episode of CL? Or ML with a previous episode of ML?**

**Response :** *This variable is qualitative and discrete in the database (“Previous history of Leishmaniasis ? Yes or No) without further information. From source documents in France we suspect that the majority of these case related to CL with previous CL but unfortunately no robust quantification can be provided. The database will be corrected for future analyses.*

4. Results 1 Reviewer 2

**114, Table 1: Demographic and clinical characteristic in this population is strikingly similar to that of infected native patients probably because travellers coming to latinamerican countries are young people looking for extreme experiences and, for such reason, are going to deep forests where this zoonotic disease is endemic and where most of native patients are infected**

**Response :** *We fully agree with the Reviewer and have added the following sentence to the discussion:* Interestingly, travelers returning from South and Central America are predominantly young backpackers visiting deep forests. In terms of age and lesion characteristics, this population resembles that of young infected native patients living in areas where leishmaniasis is endemic.

5. Results 2 Reviewer 2

**114, Table 1: The low number of patients who traveled to the New World and developed mucosal lesions could be explained by the fact that several years are usually necessary between skin lesions and mucosal compromise (range 1 to 50 or more but usually 2 to 5). Do you agree or do you have another explanation? Many of your patients went to countries like Peru and Bolivia with high prevalence of mucosal disease and your results might suggest that mucous extension is not so frequent.**

**Response:** *As commented in the discussion, proactively detecting subsequent mucosal relapse through an extended follow-up was beyond the immediate scope of our study. However, as most teams involved in the LeishMan consortium are reference centres for leishmaniasis in their respective country, they are expected to attend most patients with secondary mucosal involvement and capture corresponding information in the common database. The similar risks of mucosal involvement across our and previous reports from other teams suggest that very few if any cases of ML were missed by our consortium during the study period that lasts almost 14 years.* *Many authors of this report and specifically the 3 co-senior authors have been attending patients with CL and ML in their respective countries for more than 3 decades, drain a high proportion of (especially complex cases) to their centers, and have performed a thorough analysis of ML risk following CL (Blum et al. TMIH 2014). These experience- and evidence-based medicine approaches have led to the similar conclusion that ML is, as the Reviewer puts it with perfect accuracy, “not so frequent”. This justifies the rising message that, despite the full medical attention it deserves, mucous extension does not justify therapeutic risk at the CL stage, as systemic treatment of all CL patients may, at best, prevent a complication occurring not much more (and probably less) than 5% of patients. This complication is now usually diagnosed at the nasal stage and generally curable with 3 different drugs, namely antimony, miltefosine and liposomal amphotericin B.*

6. Results 3 Reviewer 2

**114, Table 1: "Delay from first symptoms to parasitological diagnosis (months)". Why this 2 or 4 months delay? Aren't patients looking for diagnosis? Or tests to confirm diagnosis are not easily available? It could be interesting to know which other diagnostics received before confirmation of leishmaniasis, specially because such info could be helpful for doctor with not wide experience with leishmaniasis, to keep in mind all possible differential diagnosis.**

**Response :** *Tests are available at many clinical labs in Europe but most general practitioners and dermatologists are directly confronted to a patient with CL once in their professional life-time, which explains the delay at raising leishmaniasis as a diagnosis hypothesis. The database does not capture previous diagnosis, but previous therapy which is very often simple antibiotic therapy to presumptively treat common superficial bacterial infections of the skin. We show Fig 3 that lesions form the New World generally evolve more rapidly than those for the Old World, likely explaining the difference in diagnostic delay. Backpackers travelling are also more proactive in their search for a reference center than do VFR travelling to the Old World. Awareness is important and we indicated in the discussion our hope that the clear “*clinical patterns should help attending physicians recognize typical forms of CL, hopefully reducing the diagnostic delay that currently averages 3 months”.

7. Results 4 Reviewer 2

**114, Table 1: Includes SCAR as a type of lesion could be confusing because scar is usually the final result of leish when cured. Please explain me why was included and what happen with those 4 out of 271 OW subjects reported.**

**Response :** *Physicians reported lesion type as “scar” in these 4 patients and all have a parasitological diagnosis confirmation. We investigated these cases: there were 2 children from Syria with new lesions on scar with a Leishmania recidivans diagnosis. We do not have more information on the main lesion type for the other two patients. In a few instances parasitologically confirmation was performed on a scarring lesion that had healed completely at the next consultation, which explains the final “scar” category. We changed in the table 1 “scar” with* “scar or new papule on a preexisting scar”.

8. Results 5 Reviewer 2

**135: "lymphangitis 35%" is really high. Is this "pure" leish lymphangitis or bacterial co-infections that are quite common and also produce lymphangitis?**

Response : *The rate of lymphangitis is high indeed, especially in New World CL. Lymphangitis was defined either as subcutaneous nodules in proximity to the primary lesion and/or dilated palpable lymphatic vessels in the form of a “beaded cord,” and/or regional lymphadenitis (Akilov OE Int Journal Dermatology 2007). This definition has now been added as a footnote to Table I. We cannot definitely exclude that some lymphangitis episodes were due to bacterial coinfection. However bacterial superinfection was captured independently (defined as erythematous, swollen and/or painful lesion borders and/or presence of pus) and was present in less than 10% of patients (19/218 patients; 8,7%). Compared to a typical CL-related nodular lymphangitis, bacterial lymphangitis usually displays a different clinical presentation with frequent erythema, pain and/or fever. Because physicians involved in the program were aware of the “nodular lymphangitis” variable, they were probably more proactive in their search for this clinical feature than in common practice. In many instances nodular lymphangitis was diagnosed only by palpation and had escaped the patient’s attention. In conclusion, we strongly suspect that what was observed was very predominantly “pure” leish lymphangitis, especially in patients infected in the New World.*

9. Results 6 Reviewer 2

**137, Table 2: "Duration of disease". Is this from first symptom to the consultation? Do you have info about time from the trip and first symptom? It could be useful for other doctors to know this "incubation" time to make the differential diagnosis exercise.**

Response: “*Duration of disease” corresponds indeed to time lapse between the first symptoms to the first consultation. In reality it corresponds to the duration of the disease upon first contact with the network. Because this may be counterintuitive (please see our answer to Point 13 below) we have now modified this item in Table 1 to its exact meaning i.e;* Delay from first symptoms to the first consultation. *The incubation delay as defined by the Reviewer was recently added to the new database, but (i) is difficult to estimate accurately because most travels last several months, (ii) has not been estimated in a high number of subjects because this variable is too recent.*

10. Results 7 Reviewer 2

**161: In native patients age is also a very correlated factor in mucosal disease, although most of them had initial infection (with or without cutaneous lesion) when were younger. In your over 50 patients ML was close in time to CL? In natives ML usually need a long time to appaer, what in yours?**

Response: *We found a previous history of CL (1), ML (2) or VL (2) in 5 out of 20 patients with mucosal involvement. In all cases except one (VL 20 years ago) ML occurred less than 5 years after the initial diagnosis. At this stage numbers are too small to robustly answer the reviewer’s question.*

11. Results 8 Reviewer 2

**183, Table 4: 107 patients treated with local therapies and 113 with systemic. What happen with the other 239 patients?**

Response: *Because this is a common surveillance program and not a prospective clinical trial, we could not capture follow-up data in all patients.* *Only patients with at least one follow-up with a minimum of 42 days after the beginning of treatment were included in the therapeutic analysis. This has now been clarified in the modified Fig 1 (Please see our answer to Point 1 and below).*

12. Results 9. Reviewer 2

**183, Table 4: "No specific treatment". Is this spontaneous cure? Because soap, water and dressing are good for any ulcer but 81% of cures deserve an explanation.**

Response: *As noted by the reviewer “No specific treatment” means washing the wound and applying a dressing. 81% is indeed quite a high rate of spontaneous cure but the number of patients treated with this approach was small (21 patients). These patients were selected based on previously published criteria (Morizot et al Clinical Infectious Diseases 2013). In this previous experience the “spontaneous cure rate” was even higher (92%), please see the table below extracted from Morizot et al Clinical Infectious Diseases 2013. In the present study, most patients treated with this approach (19/21) were infected in Old World, predominantly with L. major.*

*We have added the following sentence to the results section:* As previously observed “No specific treatment”, which corresponds to washing lesions with soap and water followed by applying a semi-occlusive dressing, was associated with a high (81%) cure rate (Morizot CID 2013). Patients treated with this approach were mainly infected in OW (90%; 19/21).

13. Results 10. Reviewer 3

**In the table 1, the authors shown the following results: Delay from first symptoms to parasitological diagnosis (months), median [IQR] [1-3] 4 [2-6] <0·01. Duration of disease (in months), median [IQR] 3 [2-4] 4 [3-7] <0·01. How to explain that the delay in diagnosis is longer than the time of illness?**

Response : *As indicated in our response to Point 9 above, the variable “Duration of disease” is a misnomer as it only indicates the duration of disease before seeking medical attention (not the total duration of disease). We have therefore renamed this variable in Table I to* Delay from first symptoms to the first consultation*.*

*The Reviewer is also right in pointing that delay in diagnosis in OW CL seem to be longer than the duration of the disease, which is indeed counterintuitive. This is likely explained by missing data for both variables and because both delays are very close one to the other (3 months, IQR 1-6 versus 3 months, IQR 2-6) in the total cohort. Because this is confusing we have removed the delay to parasitological diagnosis.*

14. Results 11. Reviewer 3

**The authors mention that ulcerated injuries were more frequent in travelers traveling from the new world (75.5%) than from the old world (47.5%), but there was no statistical difference. How to affirm or explain this finding?
3) Likewise, they state that the lesions are more frequently localized on the limbs (61% versus 47%) and more frequently associated with nodular lymphangitis (30% versus 6%), comparing New and Old world. there is no statistical difference presented. Please, clarify these point.**

Response : *As requested by the Reviewer, we have performed the statistical analysis for these 2 variables in OW and NW patients. Results are as follows :*

*-type of lesions between OW and NW: Chi square test. X_2_ (5, N=455)=50.50, p<0.01.*

*-lesions localization between OW and NW: Chi square test. X_2_ (6, N=457)=32.57, p<0.01.*

*We did not add these results to the initial submission because Chi square between 2 groups with categorical variables with more than 3 levels should be interpreted with caution. We have now added this statistical analysis to Table I.*

15. Results 12. Reviewer 3

**Another important point is related to treatment. The most interesting data would be to evaluate the therapeutic response in relation to the species of Leishmania. I believe that you cannot compare systemic treatment with local, if there is no well-defined parameter. For example: compare response to different treatments in patients with similar lesions.**

Response: *We agree with the Reviewer that the evaluation of therapeutic response of each regimen for each species of Leishmania would be very interesting and would facilitate an optimized management of CL. However, there are many species and many regimens. This diversity reduces the analytical power by sub-groups. We hope that the continued efforts of large networks will contribute to further analyses of correlation between clinical presentation, species, treatment and outcomes. At the moment this is beyond the scope of this manuscript.*

16. Results 13. Reviewer 4

**The most curious result was related to the mucosal involvement rate detected in patients infected with L. infantum. Also, the evidence of immunocompromise as a factor associated with such an involvement. However, the merit of the comparison of the L. infantum group against the New World acquired cases infected with parasites belonging to L. braziliensis complex should be revised. In fact, epidemiology and pathogenesis of ML associated to L. braziliensis complex is pretty different of the pattern described for the L. infantum mucosal disease. The relevant comment on that is slightly mentioned in the discussion when authors refer to the long-term follow-up needed for concluding on ML rates for patients who received local treatment. It could be misleading if readers were induced to consider that ML is more relevant for L. infantum infected patients. Instead of that, the results of this study confirm that for immunocompetent individuals ML continues to be more relevant in cases infected with parasites belonging to L. braziliensis complex. Then, an additional analysis should be done excluding the immunocompromised patients.**

Response : We agree with the reviewer that the high incidence of ML in *L. infantum*-infected patients is a new, useful information in the context of management of CL and ML in travelers. We also agree that this should not translate into premature conclusions regarding the mechanisms of mucosal involvement during or after episodes of CL. As we wrote in the discussion « *Discriminating the respective contributions of parasite-, vector- and host-related factors in these new phenotypes is beyond the scope of our current approach but the sustained, multisite, multiparametric surveys performed by the LeishMan network and other groups worldwide will provide information to more precisely revisit the pathogenesis of human leishmaniasis [9].”* As indicated under Point 2 above, the number of ML cases is currently too small to engage efficiently into subgroup analyses in the search for robust risk factors. Immunocompromised patients with immunosuppression may or may not have a higher risk of subsequent ML when attended for CL due to *L. braziliensis* or *L. guyanensis*. Future studies will clarify these important issues.

17. Results 14. Reviewer 4

**The results of the multivariate analysis for ML determinants should be summarized in a table with the unadjusted and adjusted ORs and their respective 95%CI.**

Please find below the detailed results of multivariate analysis.

The following variables: age, sex, area (New World or Old World) where infection was acquired, immunosuppression were included in the multivariable conditional logistic regression analysis. In multivariable analysis, immunosuppression (OR 10.8 IC 95% [3.4–34]), age > 50 years (OR 4.8 IC 95% [1.7–14]), and male gender (OR 3.6 IC 95% [1.05–12.6]) were independently associated with mucosal involvement.

Please see the raw tables of stepwise logistic regression in the Statview software with unadjusted and adjusted ORs (Raw tables are in French because of the software language):

Unadjusted ORs

Adjusted ORs

As discussed with *with Reviewer #4 above. We agree that multivariate approach is criticable on some points.*

*A reasonable sample size for a logistic regression model should include at least 10 events per independent variable. We only had 20 episodes of mucosal involvement that mean we can theorically include in our multivariate model: 2 variables.*

*We choose to include more variables (5) with statistical significance and biological plausibility. However, this method is largely criticable and we think that no approach including ours could bypass methodological issues of this multivariate analysis. This is largely explain by the low number of events compared to the number of patients included.*

*As no approach seem to be perfect, we choose, if the Editors agree, to not include the multivariate analysis in the definitive manuscript.*

However, as indicated under Point 2, it seems at this stage more robust to delete this multivariate analysis. We have now added this limitation to the corresponding paragraph of the discussion as follows:

This study has limitations. **The number of mucosal or mucocutaneous episodes was not large enough to enable a robust multivariate analysis of the risk factors for mucosal involvement.** Because this is a common surveillance program and not a prospective, comparative clinical trial, we could not capture follow-up data in all patients, hence the need for a careful interpretation of our observations regarding therapy. Not least, HIV testing was not systematically done which may limit our conclusions regarding the impact of immunosuppression on the clinical spectrum of cutaneous leishmaniasis. Nevertheless, this robust description of clinical, parasitological and therapeutic features of cutaneous leishmaniasis in almost half a thousand of travelers, reveals a moving landscape, where the risk of mucosal involvement is not limited to travels in the New World and effective treatments of CL are not limited to systemic therapy.

18. Results 15. Reviewer 4

**Therapeutic data were available from 220 patients. This represents a large data loss of ~50%. An explanation should be offered and this issue should be highlighted as a relevant study limitation.**

**Response:** The reviewer is right. Because this is a common surveillance program and not a prospective clinical trial, we could not capture follow-up data in all patients. Only patients with at least one follow-up with a minimum of 42 days after the beginning of treatment were included in the therapeutic analysis. This has now been clarified in the modified Fig 1 (Please see our answer to Point 1 and below) and listed as a weakness in the corresponding paragraph of the discussion as follows:

This study has limitations. The number of mucosal or mucocutaneous episodes was not large enough to enable a robust multivariate analysis of the risk factors for mucosal involvement. **Because this is a common surveillance program and not a prospective, comparative clinical trial, we could not capture follow-up data in all patients, hence the need for a careful interpretation of our observations regarding therapy.** Not least, HIV testing was not systematically done which may limit our conclusions regarding the impact of immunosuppression on the clinical spectrum of cutaneous leishmaniasis. Nevertheless, this robust description of clinical, parasitological and therapeutic features of cutaneous leishmaniasis in almost half a thousand of travelers, reveals a moving landscape, where the risk of mucosal involvement is not limited to travels in the New World and effective treatments of CL are not limited to systemic therapy.

19. Results 16. Reviewer 4

**Were all the patients tested for HIV infection? If not all the patients had an HIV test done, it would be mention also among the limitations of the study.**

Response: HIV testing was not generalized in our cohort. We added this sentence to the Method section. “*HIV testing was not systematically done* *and/or collected as variable*”. This was also listed as a study weakness in the corresponding paragraph of the discussion as follows:

This study has limitations. The number of mucosal or mucocutaneous episodes was not large enough to enable a robust multivariate analysis of the risk factors for mucosal involvement. Because this is a common surveillance program and not a prospective, comparative clinical trial, we could not capture follow-up data in all patients, hence the need for a careful interpretation of our observations regarding therapy. **Not least, HIV testing was not systematically done which may limit our conclusions regarding the impact of immunosuppression on the clinical spectrum of cutaneous leishmaniasis.** Nevertheless, this robust description of clinical, parasitological and therapeutic features of cutaneous leishmaniasis in almost half a thousand of travelers, reveals a moving landscape, where the risk of mucosal involvement is not limited to travels in the New World and effective treatments of CL are not limited to systemic therapy.

20. Results 17. Reviewer 4

**What type of immunocompromise were registered? This is a relevant information that could appear at least as a supplementary file.**

Response. We added this important information at the results section, clinical features paragraph, as follows*:* Immunodepression was reported in 22 patients and was related to ongoing therapy with anti-cancer chemotherapy (n=1), ustekinumab (n=1), methotrexate (N=5), TNFα antagonists (n=5), prolonged corticosteroids with or without other immunosuppressive drugs (azathioprine (n=1), mycophenolate mofetil (n=1), corticosteroids alone (n=4)), or HIV infection (n=3), or Good syndrome (n=1).

21. Results 18. Reviewer 4

**Finally, the standard of care could be changed during the study period and this aspect deserves at least a comment in the discussion section.**

Response: There was indeed some heterogeneity in the therapeutic management. Nevertheless, the general algorithm (as published by Morizot et al. CID 2013 Fig3) has been the backbone during the whole period.

*We added this information in discussion section :* Approximately two thirds of patients of our cohort were eligible for local therapy when following guidelines in travellers based on lesions number, size, location and preexisting conditions [7,12,15]. Even though there may have been some heterogeneity in therapeutic management during the study period, the general algorithm we described in 2013 has remained our consortium’s therapeutic management backbone.

22. Conclusion 1. Reviewer 2

**223: Yes, this comment goes in the same line with my previous (line 114) and that's why a list of initial diagnosis done before to confirm leish could be very useful for doctors with less experience in this disease.**

**Response :** Data concerning differential diagnoses before the confirmation of leishmaniasis were not recorded in the database. In our experience, superficial bacterial infection is by far the predominant differential diagnosis of leishmaniasis in travelers, a topic explored by several excellent teams and networks (see for example Caumes et al. Clinical Inf Dis 1995;20:542-8).

23. Conclusion 2. Reviewer 2

**271: "Detecting subsequent mucosal relapse through an extended follow-up was beyond the immediate scope of our study". OK, but at least you could tell us if any of the patients attended during the initial years developed ML during the time when the study was still active. My concern is because people with no big experience in CL and ML can read the paper and understand that ML appears soon after CL and forget long-term surveillance.**

**Response:***.* We have added the following sentence to the results section. “A previous history of leishmaniasis was reported in 5 of 20 patients with mucosal involvement. CL, ML or VL had been diagnosed in 1, 2 and 2 patients respectively. In all patients except one (a previous VL episode 20 years before), the mucosal involvement occurred less than 5 years (median = 4 years) after the first episode.”

We have also added the following sentence at the discussion section as a reminder for the necessity to follow patients treated for CL and inform them of the risk of delayed mucosal involvement:

In our cohort, a previous episode of leishmaniasis was reported in 20% of patients with mucosal involvement. Several teams in our consortium give to CL patients a note mentioning the diagnosis of leishmaniasis and the significant (though relatively low) risk of oro-nasal complications that may occur years to decades after the initial episode. Our results suggest that a similar information should also be provided after a VL episode.

The information presented in this manuscript will be used to help patients better grasp the significant yet relatively low risk of this complication.

24. Conclusion 4. Reviewer 3 and Reviewer 4

**Rev 3: The main conclusion of this manuscript is be immunocompromised and be over fifty years old are risk factors to develop mucosal leishmaniasis.
The authors not mentioned the limitations of manuscript.
The topic addressed in this manuscript is very important for public health, mainly to alert health professionals from non-endemic areas to leishmaniasis.**

**Rev 4: Most of the conclusions are supported by the data. The study limitations should be clearly stated as requested in above the comments. After considering the study limitations, the abstract section deserves some attention to be less emphatic for conclusions based on limited data.**

**Response :** We have now added a paragraph listing the main limitations of the study to the discussion section as follows :

This study has limitations. The number of mucosal or mucocutaneous episodes was too small to enable a robust multivariate analysis of the risk factors for mucosal involvement. Because this is a common surveillance program and not a prospective, comparative clinical trial, we could not capture follow-up data in all patients, hence the need for a careful interpretation of our observations regarding therapy. Not least, HIV testing was not systematically done which may limit our conclusions regarding the impact of immunosuppression on the clinical spectrum of cutaneous leishmaniasis. Nevertheless, this robust description of clinical, parasitological and therapeutic features of cutaneous leishmaniasis in almost half a thousand of travelers, reveals a moving landscape, where the risk of mucosal involvement is not limited to travels in the New World and effective treatments of CL are not limited to systemic therapy.

Regarding the conclusion, we amended it in the abstract and in the author summary according Rev.4 comment.

**ABSTRACT : Conclusion/Significance:** CL acquired in *L. infantum*-endemic European and Mediterranean areas displays unexpected high rates of mucosal involvement comparable to those of CL acquired in Latin America, especially in immunocompromised patients. ~~In immunocompetent patients, mucosal involvement is rare.~~ When used as per recommendations, local therapy is associated with high cure rates.

**AUTHOR SUMMARY :** …“In striking contrast with previous assumptions, the study shows that CL acquired in Europe displays unexpected high rates of mucosal involvement comparable to those of CL acquired in Latin America, especially in immunocompromised travellers. It also shows that ~~in immunocompetent travellers, mucosal spread is rare and that~~ when used as per recommendations, local therapy is associated with high cure rates.

25. Editorial 1. Reviewer 1:

**The Title “Clinical diversity, mucosal involvement and treatment strategy in cutaneous leishmaniasis: a European clinical report in 459 patients” is a little off. I think the authors mean “Clinical diversity, mucosal involvement and treatment RESULTS in cutaneous leishmaniasis: a European clinical report OF 459 patients.” Note that the title is not really accurate with respect to mucosal involvement, because it implies that all cases of mucosal involvement were seen in concomitant cutaneous disease, but I am not sure how to fix this. “Clinical diversity, mucosal involvement and treatment RESULTS in cutaneous and mucosal leishmaniasis” is redundant (of course there is mucosal involvement in mucosal leishmaniasis). “Clinical diversity and treatment RESULTS in cutaneous, mucocutaneous, and mucosal leishmaniasis:” is probably best.**

**Response:** We have changed the title to: “Clinical Diversity and Treatment Results in Cutaneous, Mucocutaneous, and Mucosal Leishmaniasis: a European Clinical Report of 459 patients “.

26. Editorial 2. Reviewer 1:

**The heading “Clinical Features” at line 122 should be “Clinical Features of Cutaneous Disease.**

**Response:** Done

27. Editorial 3. Reviewer 1:

**“ Mucosal Involvement”, the second focus of the report, is tricky. Mucocutaneous disease (mucosal disease with cutaneous disease) and mucosal disease per se (only mucosal disease) are correctly defined in Definitions, but it might be there added that mucosal involvement includes both presentations. I found it hard to track mucocutaneous disease separately from mucosal disease. In Table 2, “mucosal disease” might be replaced by “mucocutaneous disease” to make it clear that for Table 2 (cutaneous disease), all “mucosal disease” was mucocutaneous. In the paragraph beginning on line 145, the sentence “We observed 10 MCL, 9 ML, and one MCL with visceral involvement and inaugural skin lesions in a patient with AIDS (CD4 count 67/mm3)” is welcome because it separates the several presentations. It would be helpful if in the following sentences “Mouth and laryngeal lesions were observed in 6/13 cases from Old World and 1/5 case from New World, while lesions of nasal cavity were observed in 7/13 cases from Old World and 5/5 cases from New World. Seven of 15 infections (47%) with mucosal involvement in the Old World were observed in immunocompromised patients while no patient was immunocompromised in the New World subgroup with mucosal involvement”, the authors could give an idea of the breakout of mucocutaneous vs mucosal disease. In Discussion, it is said that “mucosal involvement was observed in…22% of patients infected with L. infantum, in whom this complication was strongly related to pre-existing immunosuppression.” At least this reviewer could not figure out the L infantum cases. Were these mucosally-involved cases mucosal spread from a cutaneous focus (mucocutaneous disease) or frank mucosal disease without other organs (skin, viscera) being involved? In spite of these issues, the sentence in Discussion “In our study, the unexpected, low rate of mucosal involvement in infections acquired in the New World contrasts with the unexpected, relatively high rate in infections acquired in the Old World” is both cleverly worded and accurate.**

**Response:** We have added the following sentence at the Methods section: “Mucosal involvement refers to both ML and MCL”.

We changed the title of Table 2 “Comparative features of 166 cases of cutaneous leishmaniasis by main infecting complex species*” by “*Comparative features of 166 cases of cutaneous, mucocutaneous and mucosal leishmaniasis by main infecting complex species*”. We also added type of mucosal involvement (ML or MCL) in last row of the table*

| **Mucosal involvement** | 6% (2)  ML (1)  MCL(1) | 5% (1)  MCL (1) | 0% (0) | 0% (0) | 22% (8)  ML(4)  MCL(4) |
| --- | --- | --- | --- | --- | --- |

We have added in the text this sentence to better clarify the breakout ML vs MCL: *“*Of the 5 patients from the New World 2 had ML and 3 had MCL, while of the 15 patients from the Old World 7 had ML and 8 had MCL *”*

We also changed the following sentences: **“**whereas it affected 6% of patients infected with *L. braziliensis* or *L. guyanensis complex* (2 MCL, 1 ML) and 22% of patients infected with *L. infantum* (4ML, 4MCL), in whom this complication was strongly related to pre-existing immunosuppression.”

28. Editorial 4. Reviewer 3:

**The authors mentioned that 198 species of Leishmania were identified, but in the results and discussion, there are references to only 166 isolates. Please correct or explain this difference.**

**Response:** There were 198 patients with identified species but we focused on 5 main infecting complex species corresponding to 166 patients. See supplementary table 6.

We also added this sentence to better explain this point. “Excluding rare species and incomplete species (supplementary table 6), we focused on 5 main infecting complex species corresponding to 166 patients (table 2)”

29. Editorial 5. Reviewer 3:

**One conclusion was missing from this manuscript, electing the main findings that contribute to current literature.**

**Response :** As mentioned above, we have added the following paragraph at the end of the discussion section : This study has limitations. The number of mucosal or mucocutaneous episodes was too small to enable a robust multivariate analysis of the risk factors for mucosal involvement. Because this is a common surveillance program and not a prospective, comparative clinical trial, we could not capture follow-up data in all patients, hence the need for a careful interpretation of our observations regarding therapy. Not least, HIV testing was not systematically done which may limit our conclusions regarding the impact of immunosuppression on the clinical spectrum of cutaneous leishmaniasis. Nevertheless, this robust description of clinical, parasitological and therapeutic features of cutaneous leishmaniasis in almost half a thousand of travelers, reveals a moving landscape, where the risk of mucosal involvement is not limited to travels in the New World and effective treatments of CL are not limited to systemic therapy.

30. Summary and General Comments. All reviewers

**Response:** We thank again the reviewers for their thoughtful comments and are honored by the time, as well as the deep and clever thoughts they have dedicated to the review process. We are very pleased and proud that English was considered of good quality. Sadly but honestly, we have to stress that English natives in the coauthors list deserve most of the credit…
