# Supplementary material for: Clinical diversity and treatment results in Tegumentary Leishmaniasis: A European clinical report in 459 patients
Source: PLoS Negl Trop Dis. 2021 Oct 13;15(10):e0009863. doi: 10.1371/journal.pntd.0009863 (PMC8544871; doi:10.1371/journal.pntd.0009863)
Supplement: Attachment — Submitted filename: 2021-09-07-Rebuttal-2.docx [file pntd.0009863.s006.docx]

Responses to Reviewer's Questions & Comments

**Methods**

Reviewer #1: Methods:good

Reviewer #2: Due to the wide diversity of species and clinical presentations but with very few cases in each specific situation, I consider that the most appropriate title should be Clinical diversity and treatment of tegumentary leishmaniasis (there is no enough information on ML to be specifically mentioned in the title).

We have now changed the title using “tegumentary” as requested. Of note, we thus come back to the initial title that had been changed according reviewer’s #1 request during the first revision round.

47 to 53: definition of CL overlaps with that of MCL: "...A patient was considered to have CL if she/he had: (1) cutaneous and/or mucosal lesions..." and "...Muco-cutaneous leishmaniasis (MCL) refers to the simultaneous presence of both mucosal and skin lesions...". I think CL is only cutaneous lesion (not "and/or mucosal").

The sentence was modified accordingly

“ A patient was considered to have cutaneous/mucocutaneous leishmaniasis if she/he had:”

77: "..., lesion type (papulo-nodular or dry crust or wet crust)..."  Not ulcers? Ulcers are the type of lesions usually treated with local therapies.

Wet crust is equivalent to ulcer in our terminology, as made explicit in Table 1.

Extract of table 1

| Type of lesions | |  | |  | | < 0.01 | |
| --- | --- | --- | --- | --- | --- | --- | --- |
| Ulcer (wet crust) | 75·5% (139/184) | | 47·5% (128/271) | |  | |  |

Reviewer #3: The objectives are intrinscially related with hypothesis. The study design is appropriate to descritive of cohort study proposed. The population is clearly described, however the sample size is not sufficient to power of the hyphotesis. For example, to estimate the prevalence or frequence of mucosal leishmaniasis,  or evaluate the response to local therapy the numeber need to be higher.
Statistical analysis support the conclusions.
There was compliance with ethical precepts. All regulations were followed.

We fully agree with reviewer #3 that our care should be taken not to overinterpret the data. The arguments put forward by the reviewer are the reasons why we have chosen not to include our multivariate analysis for the ML risk and being only descriptive for treatment evaluation.

Reviewer #4: Authors have made all the modifications requested and they also have offered the proper clarifications. Methods are adequate.

**Results**
-Does the analysis presented match the analysis plan?
-Are the results clearly and completely presented?
-Are the figures (Tables, Images) of sufficient quality for clarity?

Reviewer #1: REsults: good

Reviewer #2: 111: "... and a previous history of leishmaniasis was reported by 8% of patients in the cohort". I insist that it would be important to know if these patients had had skin or mucosal lesions as a previous episode.

This important issue was addressed in the first rebuttal letter as follows: “*This variable is qualitative and discrete in the database (“Previous history of Leishmaniasis ? Yes or No) without further information. From source documents in France, we suspect that the majority of these case related to CL with previous CL but unfortunately no robust quantification can be provided. The database will be corrected for future analyses »*

131: "...Excluding rare species and incomplete species (supplementary table 6), we focused on the 5 most frequent infecting complex species...".  Good decision.

We thank the reviewer for this comment.

197, Table 4: "Wash lesion and wound dressing" it could be apparently enough to cure OW CL at similar rates to systemic or topical therapies; so this local care could be recommended as treatment at least to be tested in a clinical trial? It could be THE treatment of many patients in the old world and some in the new world? Or, in the opinion of the authors, are these local measures an adjunctive management of a local or systemic treatment with drugs?

As mentioned by the reviewer #3 regarding the design of the study, we cannot compare wash-wound dressing/local/systemic treatment but it could be an option in selected patients. Some guidelines (Blum 2014) support this approach only in OWCL in selected patients who agree with this approach with a careful follow-up. We think that wash lesion and wound dressing are mainly an adjunctive treatment as articles with topical paromomycin show that wash and dress is significantly less effective that topical paromomycin in this context (see for example Ben Salah PLoS NTD 2009 and Ben Salah NEJM 2013).

Reviewer #3: The results are clearly presented, however some points in the table 1 (for example) is not complete.

As commented above, we present results that are robustly captured in the database. This database cannot be as precise as a database for a prospective clinical trial. Resources are limited to study tegumentary leishmaniasis.

Reviewer #4: Authors have made all the modifications requested an.d they also have offered the proper clarifications. The results section is adequate.

--------------------

Conclusions
-Are the conclusions supported by the data presented?
-Are the limitations of analysis clearly described?
-Do the authors discuss how these data can be helpful to advance our understanding of the topic under study?
-Is public health relevance addressed?

Reviewer #1: conclusions: Good

Reviewer #2: If immunocompromised patients make mucosal lesions in the old world is understandable but that new world patients with all their immune system functioning make recent or late mucosal lesions is more worrying. In fact, what we observe is that mucosal patients in the New World improve with treatment but have frequent recurrences. 

Infiltration and ulceration in tegumentary leishmaniasis is the consequence of an active host immune system fighting against parasites. Accordingly, how can mucosal presentation be explained in the immunosuppressed from the old world compared to the immunocompetent from the new world? Would the authors write a paragraph considering this aspect?

This is a very interesting point, but that would probably find its best place in a review. The paragraph would indeed be very speculative and, in this thorough reviewing process, we lie between a rock and a hard place, some reviewers urging us not to be speculative. Nevertheless, we have added a sentence on the fact that pathogenic processes are probably different in different continents.

“Immunosuppression seems to play an important role in the pathogenesis of ML in the Old World, while its role in ML in New World is more difficult to determine, as many apparently immunocompetent patients do develop ML

"...the risk of mucosal involvement is not limited to travels in the New World and effective treatments of CL are not limited to systemic therapy". I fully agree that these are the main findings of this paper, as the authors say in the added paragraph in which they mention the limitations of the study.

We are grateful to the reviewer for this comment.

Reviewer #3: The conclusions are supported by the results and the limitations are described.
The manuscript present relevant data for public health

Reviewer #4: Authors have made all the modifications requested and they also offered the proper clarifications. The discussion and conclusions sections are adequate.

--------------------

Editorial and Data Presentation Modifications?
Use  this section for editorial suggestions as well as relatively minor modifications of existing data that would enhance clarity. If the only modifications needed are minor and/or editorial, you may wish to recommend “Minor Revision” or “Accept”.

Reviewer #1: (No Response)

Reviewer #2: With the modifications and adjustments in tables and texts it becomes clearer

Reviewer #3: It is necessary rewrite the table 1, containing information about nodular lymphangitis

Nodular lymphangitis is indeed an interesting item both clinically and in terms of pathogenesis. However, as mentioned above, we can only present results that are robustly captured in the database. As it stands Table 1 is very comprehensive and contains all major and robust items. Its last line contains the incidence of nodular lymphangitis in each group.

Reviewer #4: None

--------------------

Summary and General Comments
Use  this section to provide overall comments, discuss strengths/weaknesses of the study, novelty, significance, general execution and scholarship. You may also include additional comments for the author, including concerns about dual publication, research ethics, or publication ethics. If requesting major revision, please articulate the new experiments that are needed.

Reviewer #1: Well revised

Reviewer #2: Paper improved with changes in text and tables. The limitations are now more evident to the reader and allow him/her to read with a better perspective.

Reviewer #3: The authors present comparing tegumentary leishmaniasis from the Old and New World. The topic is quite interesting and current and really requires more information in the literature to support decision-making in public health. However some point need to be improved. Bellow I send my considerations and suggestions.
1. Abstract
\# In table 1, the authors present data on the size of the lesion in mm. I suggest keeping the same unit as shown in table 1 (mm) or leaving cm in the table.

The size of lesion has been harmonized in mm across the manuscript.

\# The authors refer that in the old world there was 5% of mucosal involvement, however, in table 1, the prevalence of mucosal damage was between 2 and 2.5%. Please review this information. It is unclear.

As mentioned in the table 1, we are not talking of mucosal involvement but clinical forms. When pooling mucocutaneous forms (2,5%), (pure) mucosal forms (2,5%) and MCL-VL lesions, you obtain 5,3% of mucosal involvement in OW corresponding to 15 patients.

Extract of table 1

| Muco-cutaneous | 2% (3) | 2·5% (7) |
| --- | --- | --- |
| Mucosal | 1% (2) | 2·5% (7) |
| Muco-cutaneous and visceral leishmaniasis | 0% (0) | 0·3% (1) |

2. Mehtodology
#It is not clear whether all patients with skin lesions were evaluated for the presence of mucosal lesions, as there may be concomitant lesions. If they were evaluated, how was this evaluation done. This information can vary the frequency of mucous involvement, described in this manuscript.

This was as reported by the attending physician, so usually based on complete questioning and physical examination before and after therapy.

#The authors report a low association with immunodeficiency, however not all patients were tested for HIV. How to state this?
It would be interesting to describe how many were tested for HIV, to try to make a prevalence of co-infection in those who were tested.

In our practice, patients with CL are at very low risk of HIV infection so, as per professional recommendations, testing was usually not performed unless risk factors were identified. In Europe the prevalence of HIV infection in subjects without risk factors is < 0.1%.

#Regarding the methodology for identifying Leishmania species, was there a standard methodology used in all countries or did each center have its own methodology?

Each center has its own validated methodology. This was indicated in the method section “Information regarding the infecting *Leishmania* species was captured in the case report form by the expert based on the molecular identification performed by local laboratories in each centre or by national reference laboratories”

3. Results
\# In table 1 there is an asterisk referring to lymphangitis, however there is no lymphangitis data in the table. In the text, the authors described nodular lymphangitis (30% and 6%). Please review this data.

The asterisk corresponds to a footnote of table 1. Cf extract of table 1

| Nodular lymphangitis* | 30% (56) | 6% (16) | <0·01 |
| --- | --- | --- | --- |

Notes.

*Nodular lymphangitis was defined as subcutaneous nodules in proximity to the primary lesion and/or dilated palpable lymphatic vessels in the form of a “beaded cord,” and/or regional lymphadenitis

Univariate analysis. Categorical variables not included in the univariate analysis (indicated p-value as “NA”). Data are % (n) unless indicated.

Abbreviations: IQR, interquartile range; PKDL, Post Kala-azar Dermal Leishmaniasis.

#Out of 459 patients, only 198 had identification of the Leishmania species. In the others it was not tried to identify or was it not possible to identify? Please make this information as clear as possible.

Often not tried because diagnosis was confirmed by histology or smear, i.e., samples on which identification cannot be performed. Yet 43% is a fairly high identification rate in a program very close to current medical practice.

We added a sentence concerning this topic in the results section :

“For patients with no species identification, diagnosis was mostly confirmed by histology or smear, i.e., samples on which identification cannot be performed.”

#In table 2, the authors list the Leishmania species identified, with different variables. Regarding the New World, they describe 12 from Peru, as L. braziliensis, and 5 from French Guiana. Regarding L. guyanensis, the authors describe 8 samples from Costa Rica and 7 from French Guiana. Were the other 25 samples from Peru, 20 from Costa Rica and 29 from French Guiana not identified? This information is essential, as in French Guiana there is a prevalence of L. guyanensis and in Costa Rica there is L. infantum causing atypical cutaneous leishmaniasis.

As pointed by the reviewer, we just indicated the three more prevalent countries for the main five complex species in order to avoid too much complexity in the manuscript. For the reader who wants more precision full dataset will be available on-line.

#In table 3, the authors compare the number of lesions between mucous involvement and without mucosal involvement. Was this analysis performed only on patients who had cutaneous and mucosal lesions? Please make this information clearer.

The analysis was performed in all patients with mucosal involvement as mentioned in the result section: “We observed 10 MCL, 9 ML, and one MCL with visceral involvement and inaugural skin lesions in a patient with AIDS (CD4 count 67/mm3).”

#In table 4, the authors compare local and systemic treatment in old world patients. However, I believe that there may be a bias, since the indication for treatment is based exclusively on the characteristics of the lesion and the number of lesions.

This point is addressed in the discussion as follows: “Compared to patients who received systemic therapy, patients treated with local therapy were more frequently infected in the Old World, had smaller lesions, and less frequent nodular lymphangitis. Arguably, these differences reflect predominantly treatment choices by physicians, based on the continent of infection and, to a lesser extent, on the applicability of local treatment.”

#Regarding treatment, it would also be interesting to analyze the therapeutic response to the species, as some species are known to respond better to certain drugs than others.

This point had been raised previously. This will be performed latter when more patients are included in our surveillance program.

Discussion
#From 216 to 220 pages the author mentioned "In particular, no mucosal involvement was observed in patients infected with either L. major or L. tropica, whereas it affected 6% of patients infected with L. braziliensis or L. guyanensis complex (2 MCL, 1 ML) and 22% of patients infected with L. infantum (4 ML, 4 MCL), in whom this complication was strongly (though not exclusively) related to pre-existing immunosuppression."
I suggest review this affirmation. I believe there is a bias here, as patients who came from the New World had skin lesions. There is no reference for evaluating mucosal lesions in these patients. Furthermore, the mucosal form in the new world usually occurs after years of primary infection, mainly caused by L. braziliensis.

Mucosal involvement was reported by the attending physician, so usually based on complete questioning and physical examination before and after therapy.

This point had been raised during the previous cycle of review. We answered as follows. “*As commented in the discussion, proactively detecting subsequent mucosal relapse through an extended follow-up was beyond the immediate scope of our study. However, as most teams involved in the LeishMan consortium are reference centres for leishmaniasis in their respective country, they are expected to attend most patients with secondary mucosal involvement and capture corresponding information in the common database. The similar risks of mucosal involvement across our and previous reports from other teams suggest that very few if any cases of ML were missed by our consortium during the study period that lasts almost 14 years.* *Many authors of this report and specifically the 3 co-senior authors have been attending patients with CL and ML in their respective countries for more than 3 decades, drain a high proportion of cases (especially complex cases) to their centers, and have performed a thorough analysis of ML risk following CL (Blum et al. TMIH 2014). These experience- and evidence-based medicine approaches have led to the similar conclusion that ML is, as the Reviewer puts it with perfect accuracy, “not so frequent”. This justifies the rising message that, despite the full medical attention it deserves, mucous extension does not justify therapeutic risk at the CL stage, as systemic treatment of all CL patients may, at best, prevent a complication occurring not much more (and probably less) than 5% of patients. This complication is now usually diagnosed at the nasal stage and generally curable with 3 different drugs, namely antimony, miltefosine and liposomal amphotericin B.”*

Reviewer #4: None

--------------------

PLOS authors have the option to publish the peer review history of their article (<a href="<https://journals.plos.org/plosntds/s/editorial-and-peer-review-process#loc-peer-review-history>" target="_blank">what does this mean?</a>). If published, this will include your full peer review and any attached files.<br><br>

If you choose “no”, your identity will remain anonymous but your review may still be made public.<br><br>

<b>Do you want your identity to be public for this peer review?</b> For information about this choice, including consent withdrawal, please see our <a href="<https://www.plos.org/privacy-policy>" target="_blank">Privacy Policy</a>.

Reviewer #1: No

Reviewer #2: Yes: jaime soto

Reviewer #3: No

Reviewer #4: Yes: Gustavo Romero


Figure Files:
While revising your submission, please upload your figure files to the Preflight Analysis and Conversion Engine (PACE) digital diagnostic tool, [https://pacev2.apexcovantage.com](https://pacev2.apexcovantage.com/). PACE helps ensure that figures meet PLOS requirements. To use PACE, you must first register as a user. Then, login and navigate to the UPLOAD tab, where you will find detailed instructions on how to use the tool. If you encounter any issues or have any questions when using PACE, please email us at [figures@plos.org](mailto:figures@plos.org).

Data Requirements:
Please note that, as a condition of publication, PLOS' data policy requires that you make available all data used to draw the conclusions outlined in your manuscript. Data must be deposited in an appropriate repository, included within the body of the manuscript, or uploaded as supporting information. This includes all numerical values that were used to generate graphs, histograms etc.. For an example see here: <http://www.plosbiology.org/article/info%3Adoi%2F10.1371%2Fjournal.pbio.1001908#s5>.

Reproducibility:
To enhance the reproducibility of your results, we recommend that you deposit your laboratory protocols in [protocols.io](http://protocols.io/), where a protocol can be assigned its own identifier (DOI) such that it can be cited independently in the future. Additionally, PLOS ONE offers an option to publish peer-reviewed clinical study protocols. Read more information on sharing protocols at <https://plos.org/protocols?utm_medium=editorial-email&utm_source=authorletters&utm_campaign=protocols>

References
Please review your reference list to ensure that it is complete and correct. If you have cited papers that have been retracted, please include the rationale for doing so in the manuscript text, or remove these references and replace them with relevant current references. Any changes to the reference list should be mentioned in the rebuttal letter that accompanies your revised manuscript. If you need to cite a retracted article, indicate the article's retracted status in the References list and also include a citation and full reference for the retraction notice.
